# Short and Long-Term Outcomes After Surgical Procedures Lasting for More Than Six Hours

**DOI:** 10.1038/s41598-017-09833-7

**Published:** 2017-08-23

**Authors:** Natalia Cornellà, Joan Sancho, Antonio Sitges-Serra

**Affiliations:** Department of Surgery, Hospital del Mar, Barcelona, 08003 Spain

## Abstract

Long-term all-cause mortality and dependency after complex surgical procedures have not been assessed in the framework of value-based medicine. The aim of this study was to investigate the postoperative and long-term outcomes after surgical procedures lasting for more than six hours. Retrospective cohort study of patients undergoing a first elective complex surgical procedure between 2004 and 2013. Heart and transplant surgery was excluded. Mortality and dependency from the healthcare system were selected as outcome variables. Gender, age, ASA, creatinine, albumin kinetics, complications, benign *vs* malignant underlying condition, number of drugs at discharge, and admission and length of stay in the ICU were recorded as predictive variables. Some 620 adult patients were included in the study. Postoperative, <1year and <5years cumulative mortality was 6.8%, 17.6% and 45%, respectively. Of patients discharged from hospital after surgery, 76% remained dependent on the healthcare system. In multivariate analysis for postoperative, <1year and <5years mortality, postoperative albumin concentration, ASA score and an ICU stay >7days, were the most significant independent predictive variables. Prolonged surgery carries a significant short and long-term mortality and disability. These data may contribute to more informed decisions taken concerning major surgery in the framework of value-based medicine.

## Introduction

Life expectancy in Spain ranks fifth in the world currently according to the World Health Organization^1^. The magnitude of life expectancy improvement, however, has decreased over the last three decades during which just five additional years have been gained^2^ despite health care expenses having more than doubled: In 1995 the Spanish public expense *per capita* was 634€ and it increased up to 1408€ during the ensuing 20 years^3^. Most of this increase can be attributed to specialized and advanced care. A similar stagnation trend is being observed in other countries in the western world. For the first time, France has reported a reduction in life expectancy at birth in 2014^4^ dropping from the 3^rd^ to the 11^th^ post. This suggests that medical interventions –despite amazing sophistication of imaging, pharmacological and surgical innovations- are having a minimal impact on our populations’ health.

This paradoxical situation calls for a reappraisal of the aims and long-term outcomes of medical and surgical care and for reorientation of health care policies, particularly in those countries where medical care is financed through the public budget. Value-based medicine represents a new conceptual framework to properly assess the benefits and cost-utility of medical interventions. It has been defined as “the practice of medicine based upon the patient value and financial value associated with healthcare interventions”^5^. Value-based medicine puts special emphasis on the cost-utility of medical expenses and on the value patients get from medical interventions.

Complex surgery implies a substantial resource investment in high-risk procedures that require advanced technology and prolonged operating room occupancy. It is often performed in elderly patients with malignant conditions or requiring difficult orthopaedic or vascular reconstructions. The question arises of whether resources are appropriately allocated in terms of short- and long-term survival and disability.

Davenport *et al*. have shown that preoperative risk factors and associated morbidity explain better hospital costs than postoperative complications *per se* ^6^. Furthermore, postoperative complications not only influence 30-day mortality but may influence also long-term outcomes. Khuri *et al*.^7^ have reported that the adverse effect of a postoperative complication on patient survival was sustained even when patients who did not survive for 30 days were excluded from the analyses. Thus, future research on surgical outcomes from complex procedures should focus preferentially on their influence on life expectancy, quality of life and cost-utility.

There is no information on the potential impact of lengthy surgical procedures on long-term clinical outcomes. Thus, the present study was designed to investigate the impact of medical comorbidities on the short and long-term outcomes of surgical procedures lasting for over six hours. Surgery of long duration was chosen as a proxy for surgical complexity since it implies an operating room occupancy involving a whole 8-hour shift of surgeons, anaesthesiologists and operating theatre nurses, and a significant expense on surgical technology such as endoscopic instruments, blood savers, haemostatic devices, navigation systems, and prosthetic materials.

## Results

### Study population

**Figure 1:**
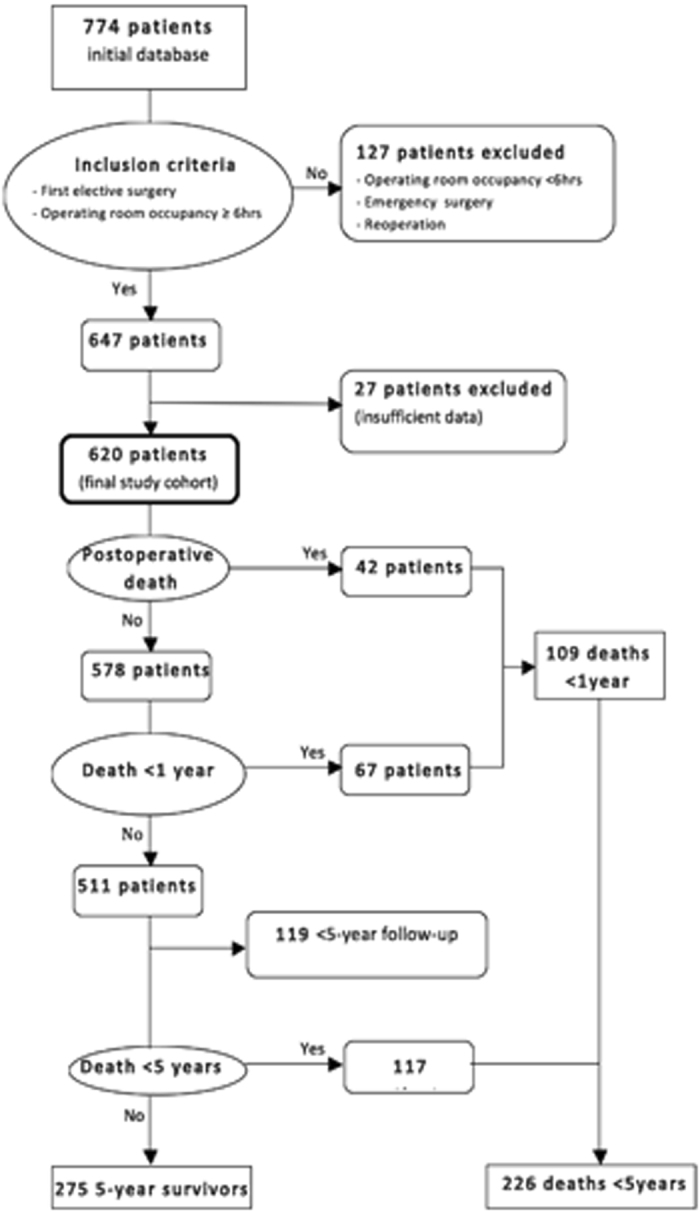
Flow chart of patients included in the study.

Some 620 patients were included (Fig. 1). Demographic and clinical characteristics are shown in Table 1. The mean operating time was seven hours. Specialties involved were Digestive Surgery (42% per cent), Gynecology (13.5 per cent), Neurosurgery (11 per cent), Vascular (10.5 per cent), Orthopaedics (8.2 per cent), Urology (8.3 per cent), Otolaryngology (2.4 per cent), Plastic (2.1 per cent) and Thoracic (1.3 per cent). Postoperative, 1-year and 5-year mortalities were 6.8, 17.6 and 45 percent, respectively. Cancer was the indication for surgery in 70% of the patients. All-cause mortality in this subgroup was similar to that of patients operated on for benign conditions within one year of surgery; it became significantly higher only after five years (50% *vs* 33%; OR 2.1C.I. 1.4–3.2; P < 0.001). Up to 75 per cent of the surviving patients developed dependency.

**Table 1:** Demographic, clinical and outcome characteristics of the study cohort (N = 620).

Study variable	Description
OR occupancy (min.)	421 ± 59
Age (yrs.)	60.4 ± 15.5
Gender (M/F)	360/260 (58%/42%)
s-Alb pre (g/L)	40.8 ± 6.2
s-Alb post (g/L)	29.9 ± 8.9
∆s-Alb (g/L)	−10.8 ± 7.2
Creatinin (mg/dL)	0.92 ± 0.41
Cancer	431 (70%)
Reoperation	112 (18%)
ICU stay > 7 days	83 (13.4%)
ICU days	2 (1–3)
LOS days	13 (9–27)
Patients with complications	476 (77%)
Postoperative death	42 (6.8%)
N drugs at discharge	6.3 ± 3
All deaths <1 year	109 (17.6%)
All deaths <5 years	226/501 (45%)
Dependency	442/578 (76%/24%)*

*Postoperative deaths excluded.

### Postoperative and 1-year mortality

Some 42 patients (6.8 per cent) died after surgery. Variables associated with death during the initial hospital admission are shown in Table 2.

**Table 2:** Variables associated with postoperative death and one year mortality in complex surgical patients.

	Postoperative death			All deaths < 1year		
	Yes (n = 42)	No (n = 578)	P	Yes (n = 109)	No (n = 511)	P
Age (yrs.)	66.9 (13.5)	59.9 (15.6)	0.005	65.8 (14)	59.3 (15.7)	<0.001
Gender (M/F)	28/14 (67%/33%)	332/246 (57%/43%)	0.242	72/37 (66%/34%)	288/233 (56%/44%)	0.04
ASA score	2.83 (0.5)	2.41 (0.6)	<0.001	2.75 (0.6)	2.37 (0.6)	<0.001
s-Alb pre (g/L)	35.2 (7.3)	41.2 (6)	<0.001	37.4 (6.3)	41.5 (5.9)	<0.001
s-Alb < 35 g/L (y/n)	17/25 (40%/60%)	82/496 (14%/86%)	<0.001	33/66 (30%/61%)	65/446 (13%/87%)	<0.001
s-Alb post (g/L)	19.5 (5)	30.7 (8.6)	<0.001	23.8 (6.4)	31.2 (9)	<0.001
∆s-Alb (g/L)	−15.71 (7.1)	−10.51 (7.1)	<0.001	−13.5 (0.7)	−10.3 (7.2)	<0.001
Creatinine pre (mg/dL)	1.1 (0.7)	0.9 (4.1)	0.003	1.1 (0.7)	0.9 (0.3)	0.007
Cr > 1.2 mg/dL (y/n)	9/33 (21%/79%)	43/535 (7%/93%)	0.002	19/89 (17%/82%)	33/478 (7%/93%)	<0.001
ICU days*	18 (4–24)	2 (1–3)	<0.001	3 (1–16)	2 (1–3)	<0.001
LOS days*	24 (8–45)	13 (9–25)	0.060	20 (9–40)	13 (9–24)	0.004
N drugs at discharge				7.2 (3.1)	6.2 (3)	0.015
Reoperation required (y/n)	13/29 (31%/69%)	99/479 (17%/83%)	0.024	21/88 (19%/81%)	91/420 (18%/82%)	0.7
Complications (y/n)	42/0 (100%/0%)	434/144 (75%/25%)	<0.001	92/17 (84%/16%)	384/127 (75%/25%)	0.03

^*^IQR (interquartile range).

Patient that died were older, had a worse ASA score and lower preoperative serum albumin concentration. Furthermore, they exhibited a very significant drop of serum albumin concentration after surgery. Multivariate analysis identified ASA score, postoperative serum albumin concentration and prolonged ICU stay as the most significant independent variables predicting postoperative death (Table 3).

**Table 3:** Multivariate analysis (binary logistic regression) of predictive variables of postoperative and long-term mortality.

	% prediction	Variables	Wald	OR	CI (95%)	P
Postoperative death	94.2%	s-Alb post (g/L)ICU stayASA Score	28.422 28.314 7.917	0.820 7.764 2.900	0.763–0.882 3.650–16.518 1.381–6.087	0.000 0.000 0.005
Mortality < 1 year	83.9%	s-Alb post (g/L)ICU stayASA ScoreCreatinin (mg/dL)N of reoperations	28.450 12.673 11.206 8.101 5.114	0.911 2.928 2.118 1.065 0.564	0.880–0.943 1.621–5.290 1.365–3.286 1.020–1.113 0.343–0.926	0.000 0.000 0.001 0.004 0.024

There was an association between preoperative hypoalbuminaemia (<35 g/L) and the prevalence (92 vs 74 per cent; P < 0.001) and severity of postoperative complications.

**Figure 2:**
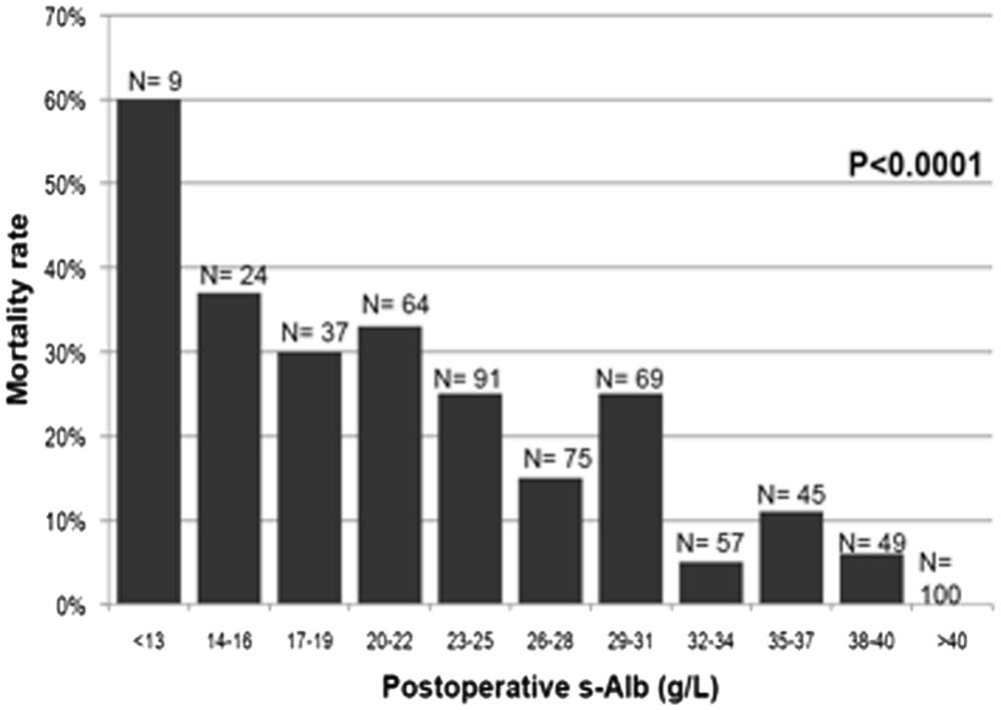
Association between all-cause mortality within one year of surgery and postoperative albumin concentrations.

Mortality within one year of surgery was 17.6 per cent (Table 2) implying that an additional 11 per cent of patients died within one year after hospital discharge. These patients were older and had worse comorbidity indices (ASA score, albumin, creatinine, number of drugs at discharge) than those surviving after one year. There was a close association between postoperative serum albumin concentrations and death from all causes within one year of surgery (Fig. 2).

### Five-year mortality

Table 4 shows the comparative analysis between patients dying and those surviving after at least five-year follow-up, namely, those patients operated between 2004 and 2011. All-cause mortality in this group was 226/501 (45 per cent).

**Table 4:** Variables associated with of all-cause mortality in 501 complex surgical patients followed for at least five years.

	Death within 5 years		P
	Yes (n = 226)	No (n = 275)	
Age (yrs.)	65.3 ± 13	56.7 ± 16.6	<0.001
Gender (M/F)	146/80 (64%/36%)	153/122 (56%/44%)	0.042
ASA score	2.6 ± 0.6	2.3 ± 0.6	<0.001
s-Alb pre (g/L)	38.6 ± 6.6	41.7 ± 5.6	<0.001
Hypoalbuminemia *s*-*Alb pre* < 35 g/L	57/169 (25%)	30/245 (11%)	<0.001
s-Alb post (g/L)	25.3 ± 6.7	32.1 ± 8.8	<0.001
∆s-Alb (g/L)	−13.3 ± 6.7	−9.6 ± 7.3	<0.001
Creatinine (mg/dL)	1.0 ± 0.6	0.9 ± 0.3	0.069
ICU days*	3 (1–6)	1.5 (1–3)	<0.001
LOS days*	17 (9–35)	12 (8–20)	<0.001
N drugs at discharge	7.1 ± 2.6	5.6 ± 3	<0.001
Reoperation	49 (21%)	40 (14.5%)	0.037
Complications	185 (82%)	199 (72%)	0.012

^*^IQR (interquartile range).

Multivariate analysis (Table 3) selected age, ASA score and postoperative serum albumin concentrations as independent variables with significant impact on long-term mortality. Thus, both ASA score and postoperative serum albumin were not only independent predictors of early death but also of death occurring long time after lengthy surgery. Mortality was unevenly distributed among surgical specialties (P < 0.001) but type of surgery was not an independent variable.

### ASA score and serum albumin

**Figure 3:**
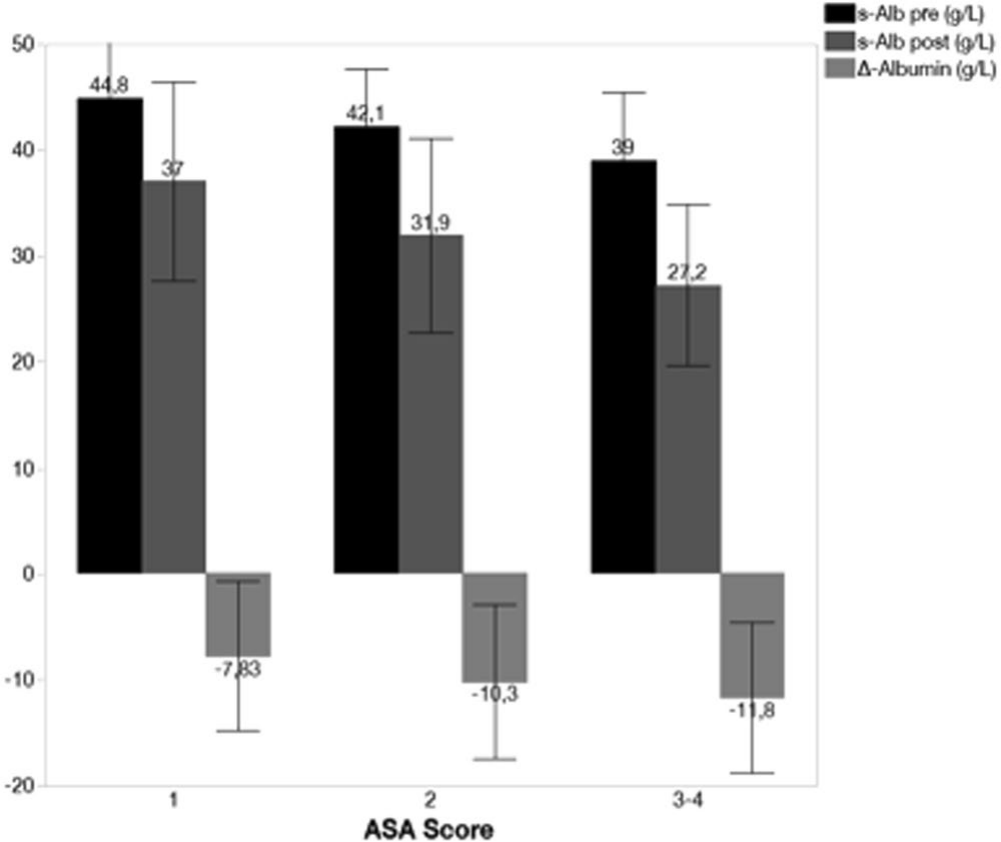
Serum albumin concentrations (pre-, post- and drop) in different ASA categories.

Postoperative mortality did correlate with the ASA score: 0 per cent for ASA 1, 3.4 per cent for ASA 2 and 10.8 per cent for ASA 3–4 (P < 0.001). This association persisted for all-cause mortality within one (3.3, 9.8 and 26.4 per cent, respectively, P < 0.0001) and five years (14.2, 38.6 and 62.1 per cent, respectively, P < 0.0001) after surgery. Interestingly, the higher the ASA score the lower pre and postoperative albumin concentrations were recorded. Drop of serum albumin concentration after surgery was more pronounced in patients with the worse ASA scores. This may explain the weak correlation (r^2^ = 0.361; p < 0.001) between the pre and postoperative serum albumin concentrations (Fig. 3).

### Age, ICU stay and death

Age was a good all-time predictor of death and dependency. The group of elderly (>70 years) patients that required an ICU stay for more than one week had a worse short- and long-term prognosis compared to those admitted for less than a week, showing a higher postoperative mortality (45 vs. 4 per cent, P < 0.001) and higher 5-year mortality 85 vs. 56 per cent; P < 0.001). Almost all (90 per cent) survivors aged >70 years became dependent on the healthcare system.

### Dependency

Over 75 per cent of the 578 patients discharged after surgery became dependent. Age, male gender, pre and postoperative albumin concentrations, need for reoperation and number of drugs at the time of hospital discharge were significant (P < 0.001) predictors of postoperative dependency. The rates of dependency increased as the ASA score worsened: 20 per cent for ASA 1, 70 per cent for ASA 2 and 90 per cent for ASA 3–4 (P < 0.001).

## Discussion

Value-based medicine is gaining acceptance as a means to assess the net benefit form medical interventions not only in terms of immediate survival, but also in terms of long-term outcomes, quality of life and cost-benefit. It is proposed as a new assessment tool because the increasing financial demands on the healthcare system driven by technological innovations, new developments in the pharmacological and imaging areas and industrial pressure, often do not translate into improvements of health indices. Some examples drawn from different areas of surgical care follow to illustrate this paradox.

1. Ten-year survival from pancreatic, gastric, lung or brain malignancies has remained almost identical at a low 1–10% for the last 40 years despite massive financial resources have been invested into their treatment and into basic and clinical research^8^.
2. Despite pleas to implement new expensive technologies in simple cholecystectomy, requiring a steep learning curve, clinical outcomes are similar or even worse using sophisticated surgical approaches (NOTES, single-port, robotic) than those obtained by standard laparoscopy^9^ or through a small subcostal incision^10^.
3. Screening for colonic cancer results in more patients being diagnosed with this malignancy and a marginal reduction of disease-specific mortality but does not extend life expectancy. Thus more colonoscopies, colectomies and chemotherapy are practiced in the screened population with no long-term value and increased costs^11^.

Along the same line, the present study indicates that a number of patients undergoing prolonged highly expensive surgery, do not obtain the expected benefit in terms of survival or personal autonomy.

Complications and death after major surgery are related to three main factors: surgical complexity, physiological limitations and comorbidity. Thus, it doesn’t seem realistic to put only emphasis on the prevention of complications^6^ because after prolonged surgery, which often combines these three major risk determinants, these will happen anyway. In our study over three quarters of patients developed at least one surgical complication, often above the Clavien-Dindo category II, and almost a fifth required a reoperation. Thus, prolonged surgery not only represents a technical challenge but also a significant physiological stress associated with the ensuing complications and reoperations. These extreme demands on the patient’s homeostasis can only be met if preoperative health is at its best, hence the relevance of serum albumin concentrations and the ASA score to predict early and late survival.

The well-known value of serum albumin as a prognostic variable^12–15^ was again confirmed in the present investigation. In addition, a significant association was found between pre- and early postoperative serum albumin concentrations and the ASA score, suggesting that serum albumin is a good indicator of both health status and the ability of the surgical patient to mount an appropriate physiological response to aggressive surgery. Multivariate analysis disclosed that postoperative serum albumin concentrations had an even greater prognostic relevance than preoperative concentrations.

Recent studies^16, 17^ have shown that serum albumin concentration on the first postoperative day after esophagectomy or pancreatectomy were the most powerful predictive variable for postoperative complications and death. Our findings confirm these reports and, in addition, reveal that postoperative serum albumin has clear-cut association with long-term survival and dependency. The drop of serum albumin concentrations after surgery is probably related to two main factors: 1) the ability of the patient to restore a normal extracellular water volume, and 2) albumin escape to the interstitial and/or third space due to lymphatic leakage or the presence of inflammatory or septic focus^18–20^. Postoperative hypoalbuminaemia has been linked to an altered distribution of the albumin molecule from the intravascular to the interstitial space by convective transcapillary transport facilitated by fluid loads^21, 22^. Thus, albumin kinetics depend to a large extent on the ability of the patient to restore the extracellular fluid volume to normal through appropriate renal and hemodynamic responses. Mullins *et al*.^23^ investigated the relationship between the fractional increase of blood volume after a rapid saline infusion and the development of complications after non-cardiac surgery. Patients with the highest increase in intravascular volume had a better postoperative course than those showing a preferential volume shift to the interstitial space. The latter experienced a more pronounced drop in serum albumin concentrations.

Multivariate analysis has been used preferentially to predict either mortality or complications in the inpatient setting^24^. Our study has extended this to mid and long-term results, showing that commonly used parameters to assess postoperative risks are also useful to predict long-term outcomes. The biological profile of patients with the highest immediate and long-term risk is the one aged 70 or more, with a low pre- and postoperative serum albumin concentration and a III-IV ASA score requiring more than a week admission in the intensive care unit, particularly if surgery was carried out for cancer. A postoperative ICU stay of over 7 days was found to be particularly lethal in the elderly as previously reported^25^. As pointed out in Pucher’s *et al*. study^26^; postoperative complications and the need for reoperation did also have an impact on long-term survival in our cohort, but at variance with this study, our findings suggest that these are weak predictive variables when compared to more stronger predictors such as ASA score, stay in the ICU and albumin kinetics.

Value-based and patient-centered surgical care implies that surgeons should pay attention to outcomes that matter most to patients. In a recent study, Berian *et al*.^27^ reported that two-thirds of patients 65 years and older undergoing inpatient surgery did loss their autonomy according to definitions close to the ones used in the present study. Loss of independency was strongly associated with increasing age, postoperative complications and the ASA score, findings that were reproduced in the present series.

In conclusion, lengthy surgery is associated with high rates of postoperative complications, short and long-term mortality and dependency from the healthcare system. A sound cost-benefit analysis should be considered when envisaging complex surgery, either for benign or malignant conditions, particularly in patients with serious comorbidities, in order to obtain the best possible outcomes from expensive and time-consuming surgical interventions.

## Patients and Methods

This is a retrospective cohort study of consecutive patients that underwent prolonged (>6 hours) first-time elective surgery at the Hospital del Mar between years 2004 to 2013. An anonymized list of such patients was provided by the Anaesthesiology Department. Patients undergoing emergency surgery, organ transplantation or reoperation were excluded. Heart surgery is not available at our institution.

### Variables under study

The following outcome variables were recorded: postoperative mortality, death from all causes within 1 year, death from all causes within five years and dependency from the healthcare system in surviving patients.

The preoperative predictive variables selected for analysis were: gender, age, ASA (American Society of Anaesthesiologists) score, disease condition (cancer, benign), creatinine and albumin. As there were only eleven ASA 4 patients, they were pooled and analyzed together with ASA 3 patients. The following postoperative variables were registered: complications as per the Clavien-Dindo classification, need and length of stay in the intensive care unit, length of hospital stay and number of drugs prescribed at the time of hospital discharge. The first postoperative (<72 hours) serum albumin concentration was recorded and its drop calculated as Δ albumin = postoperative s-alb (g/L)− preoperative s-alb (g/L).

### Definitions

#### Prolonged (complex) surgery

First-time elective surgical procedure for any specialty (except transplantation and heart surgery) lasting for more than six hours.

#### Postoperative mortality

Death during the initial hospital admission. Patients dying during readmission shortly (<30 days) after hospital discharge were also included in this category.

#### Mortality within one year

This included postoperative deaths plus all-cause mortality occurring within one year of surgery.

#### Mortality within five-years

This included postoperative deaths plus all-cause mortality occurring within five years of the initial procedure. In this category, only patients with at least 5-year follow-up (operated on up to 2010) were included.

#### Length of stay (LOS)

Total days spent in hospital since admission to discharge including early readmissions within one month after surgery.

Stay in the Intensive Care Unit (ICU): Days spent in the postoperative recovery room plus those in the intensive care unit if required. Stay in the intensive care unit was subgrouped as of short (≤7 days) or long (>7days) duration^25^.

#### Postoperative complications

classified according to the Clavien-Dindo categories^28^. For the purpose of the present study IVa and IVb categories were grouped together.

#### Dependency

Either physical disability (i.e., ostomies, blindness, paretic limb), or assistance required for daily life activities, appearing *de novo* just after surgery in patients that survived the surgical procedure; or six or more medical visits to the hospital not including visits for blood tests or radiological exams.

### Statistical analysis

An anonymized database was built from a registry kept by the Anesthesiology Department that included the length of operating room occupancy. After pruning the list according to the inclusion and exclusion criteria, outcome and predictive variables were obtained through the electronic hospital records available since 2004. For long-term follow-up, data were obtained either from hospital records or from primary care physicians.

Statistical analysis was carried out using the SPSS software package (v 21, IBM, Rochester MN, USA). For comparative analysis the Chi-square test was used for qualitative variables and the two-tailed Student’s t test and Mann-Whitney U tests for quantitative data. To assess the relevance of predictive variables at different time points, a multivariate analysis was performed (binomial regression) using as outcome variables the postoperative, 1-year and 5-year mortality. Data are expressed as proportion/percentages or as mean(SD). Statistical significance was set at *P* < 0.05.

### Ethics and regulations

This was an observational study in which chart reviews were performed using patient’s identifying number. It was not an interventional or experimental study and no specific consent forms were requested from the patients. The Hospital del Mar is a tertiary university hospital and at the time of admission patients are warned that data concerning their clinical course can be used, anonymously, for research purposes.
